# Be Smart and Active in Conservative Dentistry and Endodontics

**DOI:** 10.7759/cureus.47185

**Published:** 2023-10-17

**Authors:** Parul R Loya, Pradnya P Nikhade, Priyanka Paul, Amit Reche

**Affiliations:** 1 Public Health Dentistry, Sharad Pawar Dental College and Hospital, Datta Meghe Institute of Higher Education and Research, Wardha, IND; 2 Conservative Dentistry and Endodontics, Sharad Pawar Dental College and Hospital, Datta Meghe Institute of Higher Education and Research, Wardha, IND

**Keywords:** smart glass ionomer cement, self healing composites, smart composites, smart dentistry, smart materials

## Abstract

Numerous aspects of dentistry have been transformed by smart materials. In recent years, there have been advancements in dental materials that exhibit improved biological compatibility. These materials are specifically designed to interact effectively with the fluids found in the oral cavity, including saliva and gingival crevicular fluids. The search for the optimum restorative material results in the development of a more recent generation of dental materials known as smart materials. Smart materials react to stimuli, including stress, temperature, moisture, pH, electric field, and magnetic field, in a regulated way. Some of them are biomimetic and can imitate the dentin and enamel seen in natural teeth. These resources herald the start of a new era in dentistry known as "Smart Dentistry," and they project a promising future in terms of improved dependability and efficiency. These types of diverse materials can pick up and perform definite functionalities regarding adjustments in the nearby surroundings. Based on their capacity for recognition, analysis, and discrimination, these materials might be able to foresee problems in the near future. The superior biocompatibilities of smart materials, which have brought about a new generation of biosmart dentistry, are a crucial component of their utilization in numerous dental applications. We should use any material with intelligence as we progress in innovation and advanced technology. Additionally, we should purposefully incorporate intelligence into existing materials through design. Smart materials have proven advantageous in the field of dentistry, particularly in restorative applications. Various dental products, including smart composites, resin-modified glass ionomer materials, pit, and fissure sealants releasing amorphous calcium phosphate (ACP), smart ceramics, and compomers have all witnessed positive advancements due to the integration of smart materials.

## Introduction and background

Smart materials have been around for a while and are used in many different applications [1]. Since the 1980s, "smart" and "intelligent" have been used to describe various goods and procedures and are American in origin. During World War I, nickel was used as a sonar source in the first application of smart materials, known as magnetostrictive technologies, which assisted the Allies in locating German U-boats. Smart materials can change their properties in a controlled manner in response to stimuli like pressure, heat, moisture, pH, and magnetic or electric fields [2]. Smart materials' capability to return to their initial state even after the withdrawal of the stimulus is one of its most crucial characteristics [3]. They are known as "responsive materials" because of their great responsiveness and innate capacity to sense and respond to environmental changes [4]. A material’s self-adaptability, self-sensing, memory, and various capabilities are characterized by its smartness. In order to treat these diseases, the materials employed inside the mouth cavity should be neutral and inert. It must remain inactive and not damage the tooth's structure. Based on biocompatibility and strength, several materials are already accessible. Modern dental supplies have been improved to make them more intelligent and educated. These smart materials have changed dentistry for the better in the past few years.

## Review

Methodology

**Figure 1:**
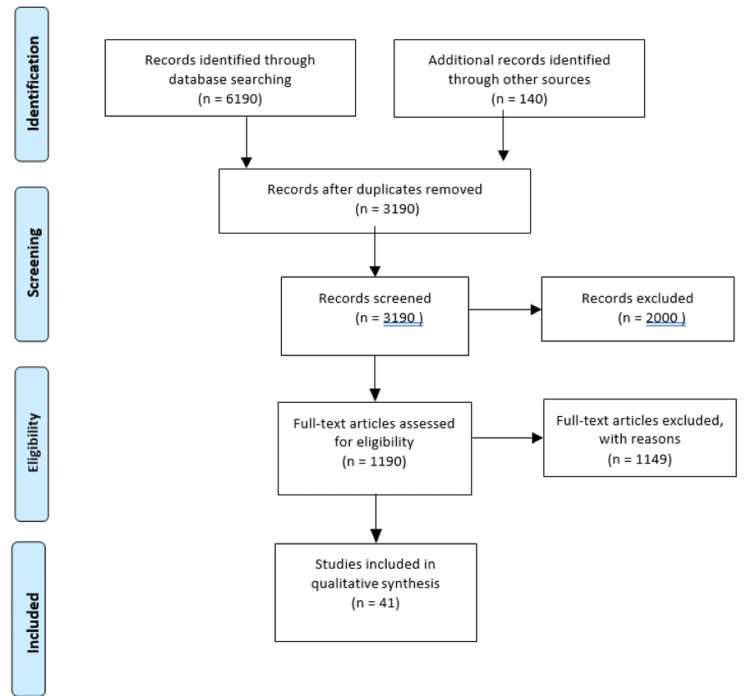
Selection process of articles used in this study Adopted from the Preferred Reporting Items for Systematic Reviews and Meta-Analyses (PRISMA) guidelines

This review carefully gathered literature on the application of smart materials utilizing electronic databases such as PubMed, Google Scholar, Web of Science, and EBSCOhost. "Smart materials", "smart dentistry", "smart glass ionomer cement", "self-healing composites", and "smart composites" were the keywords used in the search. The inclusion criteria included relevant books, articles, and reviews. The study selection procedure included screening titles and abstracts, followed by a full-text evaluation of relevant papers. The final group of included research offers a thorough analysis of the evidence that is currently available on the use of nanotechnology in dentistry. The results were combined and analyzed to draw meaningful conclusions. Figure 1 describes the selection process of articles used in our study.

Materials used in dentistry

There are different types of smart materials used in dentistry which are shown in Table 1.

**Table 1:** Smart materials used in dentistry [5]

Restorative materials	Types
1) Restorative dentistry	Smart composites, smart glass ionomer cement
2) Endodontics	NI-TI rotatory instruments
3) Prosthetic dentistry	smart impression materials, smart ceramics
4) Pediatric dentistry	Amorphous calcium phosphate (ACP) is released from pits and fissure sealants.
5) Laser dentistry	Hollow-core photonic fibers
6) Oral surgery	Smart sutures

Classification of smart materials

**Figure 2:**
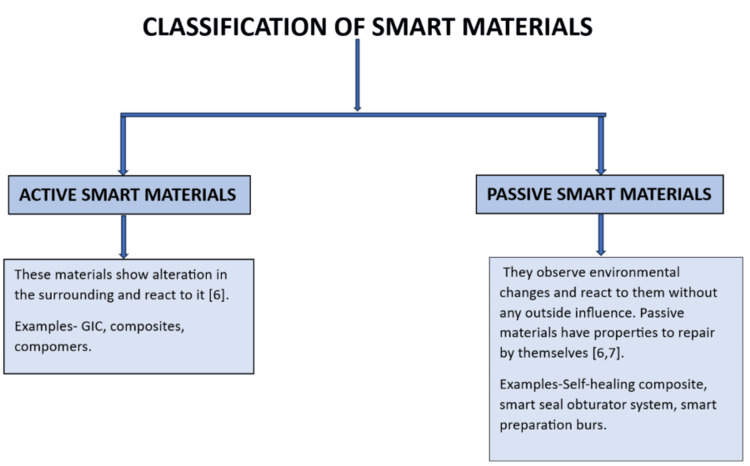
Classification of smart materials [6,7] GIC: glass ionomer cement

Smart materials are classified as shown in Figure 2.

Properties

Smart materials have thermochromic, piezoelectric, magnetorheological, shape memory, biofilm formation, photochromic, pH-sensitive, ion-release, and recharging properties. Thermochromic materials are those that respond to temperature changes by altering their color; for example, thermochromic brushes. The piezoelectric property of a material indicates mechanical tension, which results in the generation of an electric current [8]. Magnetorheological properties are seen when a material is placed in a magnetic field. Fluid materials become solid. Certain materials hold shape memory, which means that when heated, certain materials may recall their original shape after deformation, for example, Ni-Ti [9,10]. When the light conditions vary, the color of the material changes; this is called the photochromic property of a material [11]. Materials that expand or contract as the pH of the fluid around them changes, for example, smart composites containing amorphous calcium phosphate (ACP) [12], are known to be pH-sensitive. Ion release and recharging properties state that materials that initially release a large amount of fluoride can often lose their ability to continue releasing fluoride effectively over time [13-15]. However, materials containing glass ionomer cement (GIC) salt phases exhibit an intelligent characteristic. They can continuously release fluoride even after the initial burst of release, which proves to be more valuable in the long term than the rapid initial release itself. This sustained fluoride re-release is particularly significant, especially when considering the prevention of tooth decay in dental products.

Biomedical application

Recent developments in the formulation of polymers that react to specific triggers have opened up new possibilities for innovative biomedical uses. Responsive to different stimuli, these polymers can undergo alterations in their shape, surface properties, and solubility, and even form complex molecular structures and transitions between liquid and gel states. These advancements have paved the way for various fresh applications, including the fields of delivering medical treatments, tissue engineering, growing cells in a controlled environment, separating biological components, imitating biological processes, administering drugs, and producing surfaces that respond to changes in temperature [16].

Smart Pressure Bandage

Polyethylene glycols attached to different types of fibrous materials, like cotton and polyester, exhibit smart qualities like adaptability to temperature changes and undergo reversible shrinkage. This reversible shrinkage property involves giving the material the ability to remember its dimensions, causing it to contract in specific regions upon exposure to a liquid. Such materials could have practical uses in creating pressure bandages that constrict when they encounter blood [16].

Countering Radioactive Rays

Composite containers are useful for containing hazardous materials like chemical or radioactive waste. The containers are made up of fibers coated with a special substance that can detect radiation or chemicals. When these substances are detected, the coating releases other substances that can help neutralize the hazardous materials [16].

Limitations

Smart materials often pose challenges when it comes to achieving consistent stability, as they may exhibit slower response times and lower strength compared to traditional actuators and sensors. Additionally, their performance may degrade over time, making them harder to control and potentially adding to their overall cost. These are some of the limitations of smart materials.

Smart materials in dentistry

Smart Composites 

Smart composites are innovative restorative materials that contain light-activated components, alkaline substances, and nano-sized glass fillers. These composites have the ability to release specific ions like calcium, fluoride, and hydroxyl when the pH of the surrounding environment becomes lower than the critical threshold of 5.5. This function serves to protect tooth surfaces from demineralization and promote the process of remineralization [17]. Up to a 4mm thickness, they can be efficiently cured in larger quantities. Both deciduous and permanent teeth can be repaired using this technique for Class I and Class II defects. An example is Ariston pH Control introduced by the Ivoclar-Vivadent Company (Schaan, Liechtenstein)).

Amorphous calcium phosphate, one of the biologically significant calcium phosphates, is one that is most soluble and exhibits the fastest conversion to crystalline hydroxyapatite in smart composites. Amorphous calcium phosphate will operate as a source of calcium and phosphate that is effective for minimizing caries when it is incorporated into specifically designed and produced resins to create composite materials. Amorphous calcium phosphate has been examined for use in bioactive polymeric composites as a filler phase. Over time, these fibers release a significant amount of phosphate and calcium ions. Restorative materials containing the filler ACP, enclosed in a polymer binder, have the potential to promote the restoration of tooth structure [18].

At neutral or high pH levels, ACP is still amorphous calcium phosphate. Amorphous calcium phosphate undergoes changes and precipitates during a carious attack when the pH is low (at or below 5.8), replacing the hydroxyapatite (HAP) that was lost to the acid. Thus, when the pH in the mouth drops below 5.8, these ions quickly combine to form a gel. The gel transforms into amorphous crystals in two minutes, releasing calcium and phosphate ions in the process [18]. It can be said that the composites containing ACP responded intelligently to pH.

Self-Healing Composites 

Over time, materials undergo degradation due to a combination of different stressors, which could be physical, chemical, or biological in nature. As a result, the quality of the material starts to deteriorate and eventually collapse. This action leads to the release of the contained resin, which then enters the crack's space. Following this, a reaction takes place involving a Grubbs catalyst that is distributed within the epoxy composite. This reaction prompts the resin to polymerize, effectively fixing the crack and restoring the material. The potential of the self-repair mechanism relying on the disintegration of microcapsules holds great promise, and materials repaired through this method could potentially exhibit superior performance compared with macroscopic repair approaches [19].

*Smart Glass Ionomer Cement* 

Davidson was the one who initially suggested the intelligent behavior of GIC [20]. It has to attain a gel structure's capacity to quickly absorb or release solvent in response to a stimulus like a change in temperature or pH, among others. The cement mixing process conveniently measures the pores' size and number utilizing micro-computed tomography scanning [21]. Glass ionomer cement [22] has several benefits over other restorative materials, including the ability to be inserted into cavities without the use of bonding agents and having superior biocompatibility. Resin-modified glass-ionomers (RMGIs), compomers, or giomers, which comprise hydrophilic monomers and polymers like two-hydroxyethyl methacrylate (HEMA), have been introduced in order to improve the mechanical properties of standard GIC. These perceptive ions imitate the actions of human dentin. Bioactive glass (BAG) has been included in GI composition in several recent studies to increase bioactivity, have the potential for tooth regeneration and rebuilding, and reduce the occurrence of primary and recurrent caries [23].

Nickel-Titanium (NiTi) Rotary Instruments

The compound 55-Nitinol, which contains 45 weight percent titanium (Ti) and 55 weight percent nickel (Ni), is frequently used in endodontic procedures. Nitinol was first used in endodontics in 1988 by Walia et al and essentially has two phases. The austenitic or parent phase (hexagonal lattice) and the martensitic or daughter phase are both terms used to describe the high-temperature phases of a body-centered cubic lattice. Using rotary NiTi [24] files offers several benefits, including a reduced risk of file fracturing while working inside the canal, decreased operator fatigue, minimized file deviation, lower chances of canal irregularities, and limited post-operative discomfort for the patient (example: NiTi rotary files).

Nickel-titanium displays stress-induced thermoplastic behavior, where applying stress at a consistent temperature leads the austenitic crystalline phase to convert into a martensitic structure. This martensitic state renders the metal pliable and capable of bending. Upon the release of stress, the structure returns to its initial shape and enters an austenitic phase [25].

SmartPrep Burs

These are polymer burs with cutting edges that resemble a shovel. The polymer substance was created to be softer than healthy dentin but harder than carious, softened dentin. According to the manufacturer, it precisely eliminates carious dentin while leaving healthy dentin unaffected (minimally intrusive excavation); nonetheless, the cutting edges deteriorate when they come into contact with tougher materials. SmartPrep burs are only intended for one usage and come in three International Organization for Standardization (ISO) sizes: 010, 014, and 018 (self-limiting action). To avoid coming into contact with the tougher dentin, they should be used gently, and excavation should be done from the center outward. An example is the SS White diamond and carbide preparation kit [26]).

Smart Ceramics

In the past, the primary choice for dental prosthetics was porcelain-fused metal restorations. There has been a great search for good tooth-colored aesthetic restorations; thus, ceramics are extensively used as they are free from metals and have good biocompatibility, allowing them to blend properly with natural dentition. Ceramics usually consist of quartz, feldspar, kaolin, and different types of oxides [27].

Zirconia, a recent addition to the field of dental ceramics, is a versatile substance that showcases three distinct structures depending on shifts in temperature. In its natural state and around 95°C, it assumes a monoclinic crystal structure. Upon reaching temperatures beyond 95°C, zirconia undergoes a transformation into a tetragonal crystal structure. Zirconia stands as a polycrystalline ceramic, embodying the characteristics of glass ceramics. This is due to the orderly arrangement of atoms within crystalline zirconia-based ceramics, in contrast to the irregular packing observed in glass-based ceramics. Extensive evidence confirms that zirconia-based ceramics possess notably superior strength in comparison to glass-based ceramics [28].

This information indicates that the first all-ceramic tooth bridge was developed in 1995 at ETH-Zurich, Zurich, Switzerland. As a result of being introduced to the market as Cercon, the process and materials for all-ceramic tooth bridges underwent further testing. These advancements allowed for the restoration of both anterior and posterior teeth and both metal and all-ceramic abutments, as well as natural tooth preparations [29,30,31]. Excellent ease of machining and low abrasion potential are the two notable benefits it offers in contrast to enamel [32].

Smart Seal Obturation Material

The primary goal of root canal obturation is to create a secure seal within the root canal system, ensuring the prevention of additional infections or periapical issues. With the use of recent technology, there is a rising interest in simplifying the obturation process. The focus is on developing materials and techniques that can effectively fill canals with irregular shapes and minimize the formation of empty spaces, as these spaces can become breeding grounds for residual biofilms [33]. The C-point system, also known as the smart seal system, is a recently introduced method for filling root canals. It involves using a point-and-paste system that makes use of hydrophilic polymer-based technology [34,35]. The system consists of two main components: hydrophilic obturation points and a sealer. As the smart obturating material is hydrophilic, it is capable of absorbing moisture and expanding laterally to fill any voids. However, to achieve proper sealing, a sealer should be used in conjunction with these endodontic points [36,37].

They are found in a variety of tip sizes and tapers. A 6% taper is applicable to ISO tip sizes ranging from 25 to 45, while a 4% taper is used for ISO tip sizes within the 25 to 45 range. The ProTaper™ system includes tip sizes F1, F2, F3, F4, and F5. Similarly, the Sendoline™ S5 system includes tip sizes S2, S3, and S4 [38].

Smart Fibers for Laser Dentistry

The term laser refers to light amplification by stimulated radiation. The main limitations of smart lasers are their high cost and the potential for thermal injury to tooth pulp. The laser is an illustration of an electromagnetic wave generator. Three distinguishing characteristics of the laser are monochromatic, coherent, and collimated. Monochromatic elements have identical frequency and energy. Coherent waves possess a pre-established phase and are synchronized in terms of both time and velocity. Collimated beam divergence is very low, and the emitted waves are practically parallel. Applications of lasers are the analgesic effect of the laser, cavity preparation in enamel and dentin, root canal preparation, caries removal, and sinusitis. An example is hollow-core photonic crystal fibers (PCFs), neodymium yttrium aluminum garnet (Nd: YAG) [39,40,41]).

## Conclusions

The advantages of these smart materials and structures for the future are incredible. By making improvements to many products, the technology promises to offer the best solutions to extremely complex problems. It could also offer better control by reducing distortion and raising precision. Additionally, it might increase the system's preventive maintenance, which would boost its functionality. Given their numerous dental applications, there is little doubt that smart materials have a lot of potential for the future. It will be simple and convenient to correlate dental therapy with the availability of these intelligent materials, which have multipurpose capabilities, and to perform certain functions intelligently to react to changes in the immediate environment.
